# GluN2D-containing NMDA receptors in parvalbumin neurons in the nucleus accumbens regulate nocifensive responses in neuropathic pain

**DOI:** 10.1016/j.nbd.2024.106784

**Published:** 2024-12-27

**Authors:** Sukanya G. Gakare, Gajanan P. Shelkar, Dinesh Y. Gawande, Ratnamala Pavuluri, Pauravi J. Gandhi, Shashank M. Dravid

**Affiliations:** Department of Psychiatry and Behavioral Sciences, Texas A&M University School of Medicine, College Station, TX 77845, USA

**Keywords:** GluN2D, Parvalbumin interneurons, Nucleus accumbens, Cisplatin induced neuropathic pain

## Abstract

Neuropathic pain presents a significant challenge, with its underlying mechanisms still not fully understood. Here, we investigated the role of GluN2C- and GluN2D-containing NMDA receptors in the development of neuropathic pain induced by cisplatin, a widely used chemotherapeutic agent. Through genetic and pharmacological strategies, we found that GluN2D-containing NMDA receptors play a targeted role in regulating cisplatin-induced neuropathic pain (CINP), while sparing inflammatory or acute pain responses. Specifically, both GluN2D knockout (KO) mice and pharmacological blockade of GluN2D-containing receptors produced robust reduction in mechanical nocifensive response in CINP. In contrast, GluN2C KO mice behaved similar to wildtype mice in CINP but showed reduced mechanical hypersensitivity in inflammatory pain. Using conditional KO strategy, we addressed the region- and cell-type involved in GluN2D-mediated changes in CINP. Animals with conditional deletion of GluN2D receptors from parvalbumin interneurons (PVIs) or local ablation of GluN2D from nucleus accumbens (NAc) displayed reduced mechanical hypersensitivity in CINP, underscoring the pivotal role of accumbal GluN2D in PVIs in neuropathic pain. Furthermore, CINP increased excitatory neurotransmission in the NAc in wildtype mice and this effect is dampened in PV-GluN2D KO mice. Other changes in CINP in NAc included an increase in vGluT1 and c-fos labeled neurons in wildtype which were absent in PV-GluN2D KO mice. GiDREADD-induced inhibition of PVIs in the NAc produced reduction in mechanical hypersensitivity in CINP. These findings unveil a novel cell-type and region-specific role of GluN2D-containing NMDA receptors in neuropathic pain and identify PVIs in NAc as a novel mediator of pain behaviors.

## 1. Introduction

Neuropathic pain is a serious clinical problem associated with comorbidities including anxiety and depression which further affects the quality of life. Current treatment strategies such as opioids have side effects including drug dependence and may also develop tolerance. Thus, there is an urgent need to develop novel therapeutics for the treatment of neuropathic pain. NMDA receptors (NMDARs) represent rich targets for a variety of neurological disorders including chronic pain. NMDARs are tetramers composed of two GluN1 and two GluN2 subunits of which there are four types GluN2A through GluN2D. Although there is sufficient evidence for a role of GluN2B in chronic pain (Kim and Lee, 2012; Li et al., 2019; Yi-wen et al., 2021; Harris et al., 2023), the role of other subunits particularly GluN2D and GluN2C remain unclear.

The GluN2D subunit is particularly relevant to neurological disorders because of its unique biophysical and pharmacological properties. GluN2D-containing receptors are less sensitive to Mg^2+^-block, have slow deactivation, lack desensitization and have high agonist affinities (Vicini et al., 1998; Qian et al., 2005; Yamasaki et al., 2014; Hansen et al., 2018). These features allow tonic activity of these receptors and are responsible for high charge transfer. In addition, these features may lead to preferential blockade of GluN2D-containing receptors by NMDAR channel blockers particularly ketamine (Kotermanski and Johnson, 2009; Khlestova et al., 2016). Some of these features are shared by GluN2C-containing receptors including less sensitivity to Mg^2+^-block and lack of desensitization (Monyer et al., 1992, 1994; Traynelis et al., 2010) and therefore it may also contribute to tonic activity.

Here we examined the role of GluN2D as well as GluN2C subunit in nocifensive behavior in inflammatory and cisplatin-induced neuropathic pain (CINP) models. Using genetic models we found that mice with ablation of GluN2D show resistance to mechanical hypersensitivity in CINP model but are similar to wildtype mice in their response to inflammatory pain. GluN2C KO on the other hand were partially resistant to inflammatory pain but were indistinguishable from wildtype in neuropathic pain. Using conditional KO models we found that GluN2D subunit in the parvalbumin (PV)-expressing interneurons (PVIs) contribute to neuropathic pain. Furthermore, we found that accumbal GluN2D-containing receptors contribute to neuropathic pain. Moreover, the effect of GluN2D in PVIs and NAc are independent of effect on reward behaviors. Chemogenetic experiments demonstrated that PVIs in the NAc are mediator of mechanical hypersensitivity in CINP. Together these findings identify a novel mechanism underlying mechanical hypersensitivity in CINP.

## 2. Results

### 2.1. GluN2D-containing NMDAR regulates cisplatin-induced neuropathic pain

**Fig. 1.**
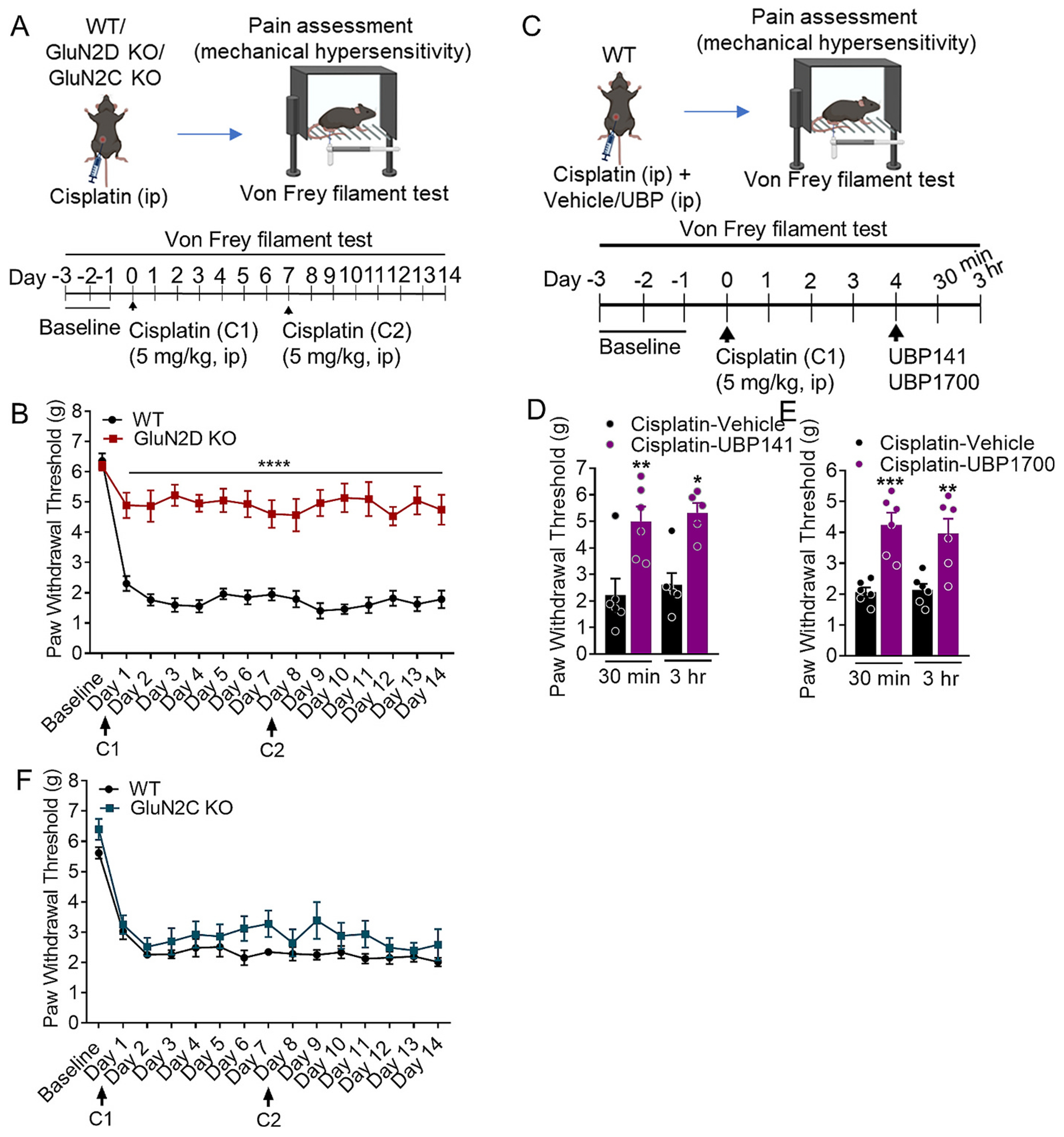
GluN2D- but not GluN2C-containing NMDA receptors (NMDARs) are resistant to cisplatin-induced neuropathic pain (CINP). (A) Experimental timeline: Schematic representation of the timeline for von Frey tests conducted at baseline and multiple time points post-cisplatin administration. (B) Effect of GluN2D ablation on mechanical hypersensitivity in CINP model. Baseline von Frey analysis indicates no significant difference in paw withdrawal thresholds between wildtype and GluN2D knockout (KO) mice. Post-cisplatin administration, wildtype mice exhibit a significant reduction in paw withdrawal thresholds, indicating increased mechanical sensitivity. In contrast, GluN2D KO mice maintain higher paw withdrawal thresholds, demonstrating reduced sensitivity to cisplatin-induced pain. Two-way ANOVA revealed significant effect of genotype, time, and their interaction (****p < 0.0001) each. (C-E) Systemic administration of GluN2D selective antagonists alleviate pain sensitivity. A significant increase in paw withdrawal threshold was observed following UBP141 (**p = 0.0063 at 30 min and *p = 0.0119 at 3 h) and UBP 1700 (***p = 0.0009 at 30 min and **p = 0.0053 at 3 h) administration in cisplatin treated wildtype mice. (F) No changes in mechanical hypersensitivity following GluN2C ablation in CINP. Data were analyzed by unpaired *t*-test or two-way ANOVA wherever appropriate. Each data point represents mean ± S.E.M.

We evaluated the role of GluN2D-containing NMDARs in the development of mechanical hypersensitivity following CINP using the GluN2D KO model. Mechanical sensitivity was tested using von Frey test at baseline and at several time points post-cisplatin administration (Fig. 1A). Baseline von Frey analysis in wildtype and GluN2D KO mice suggested no change in paw withdrawal threshold due to ablation of GluN2D subunit (Fig. 1B). Wildtype mice showed a significant reduction in the paw withdrawal threshold following cisplatin treatment. In contrast, GluN2D KO mice showed higher paw withdrawal threshold even after cisplatin administration. Significantly higher paw withdrawal thresholds were observed in GluN2D KO on days 1–14 (*p* < 0.0001, Fig. 1B) compared to wildtype mice. Two-way ANOVA showed a significant effect of genotype (F (1, 26) = 93.02, p < 0.0001), interaction (F (14, 324) = 7.676, p < 0.0001), and time (F (14, 324) = 21.09, p < 0.0001).

Further to validate results obtained in the genetic model, we used a pharmacological approach. UBP141 is a GluN2C/GluN2D preferring antagonist (Morley et al., 2005) and has been previously used at 75 mg/kg systemically (Lozovaya et al., 2014). UBP1700 is significantly more potent GluN2C/2D-preferring antagonist with nanomolar affinity (Wang et al., 2020) and was therefore used at a dose of 1 mg/kg. Pharmacological inhibition of GluN2D-containing NMDARs by systemic injection of selective GluN2D antagonists UBP141 (75 mg/kg) and UBP1700 (1 mg/kg) produced significant increase in paw withdrawal threshold in cisplatin-injected wildtype mice (Fig. 1D, 1E, respectively). One-way ANOVA showed significant effect of UBP141 (F (3, 19) = 9.313, p = 0.0005) and UBP1700 (F (3,20) = 12.33, *p* < 0.0001) treatment on the paw withdrawal threshold. Application of post-hoc Bonferroni’s test revealed a significant increase in paw withdrawal threshold following UBP 141 (*p* = 0.0063 at 30 min and *p* = 0.0119 at 3 h, Fig. 1D) and UBP 1700 (*p* = 0.0009 at 30 min and *p* = 0.0053 at 3 h, Fig. 1E) treatments.

GluN2D- and GluN2C-containing NMDARs share certain common biophysical and pharmacological properties (Traynelis et al., 2010). Therefore, to ascertain the specific impact of GluN2D-containing NMDARs in CINP, we investigated the role of GluN2C-containing NMDARs in the development of mechanical allodynia following cisplatin-induced neuropathy. GluN2C KO showed no significant difference in response to mechanical stimuli in CINP compared to wildtype (*p* > 0.05, two-way ANOVA; see Fig. 1F).

**Fig. 2.**
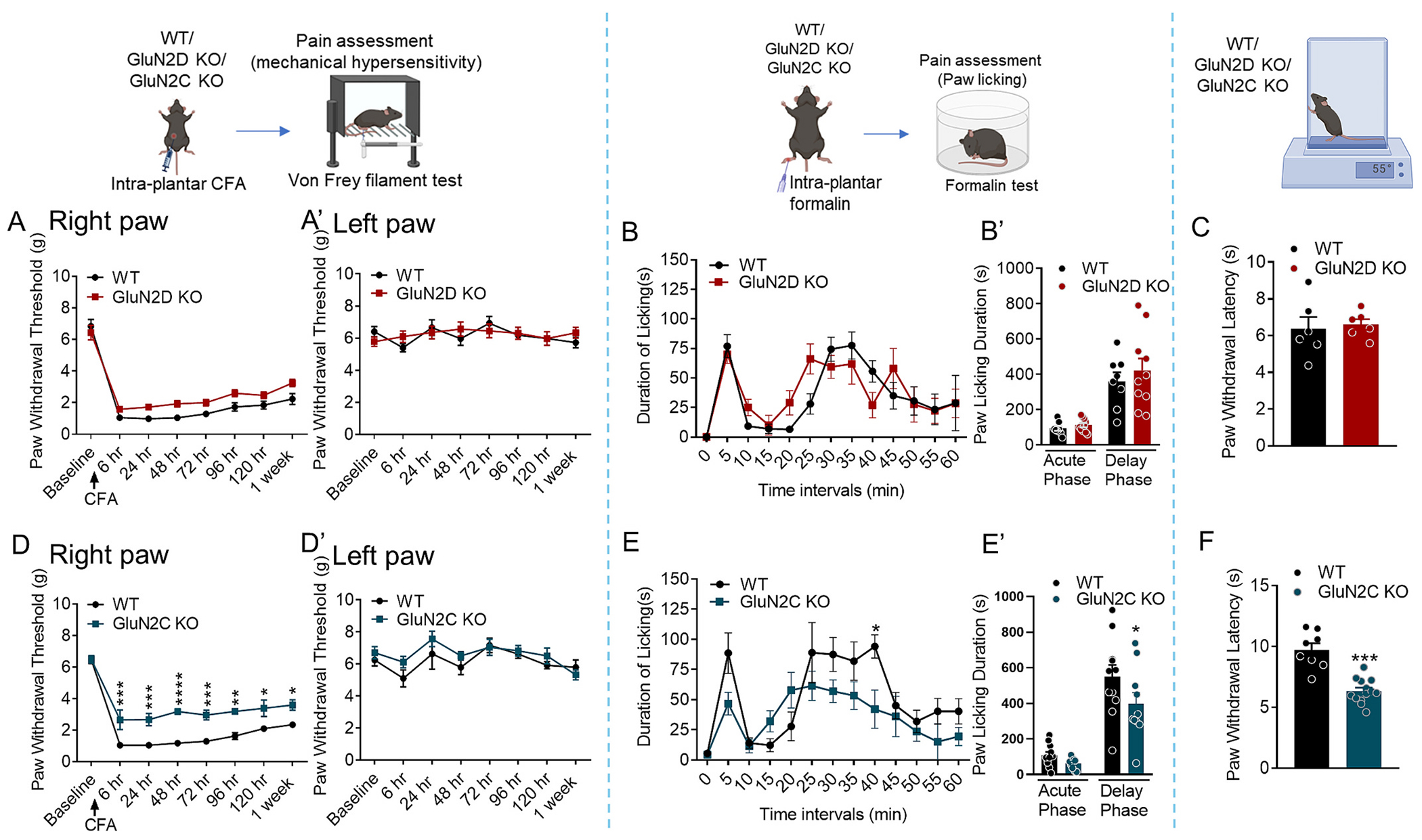
GluN2C- but not GluN2D-containing NMDARs regulate inflammatory pain responses. (A, A’) Mechanical sensitivity was assessed by von Frey test following administration of complete Freund’s adjuvant (CFA) and (B, B′) formalin in wildtype and GluN2D KO mice. No significant differences were detected between wildtype and GluN2D KO groups in both CFA-induced inflammatory condition and formalin test (*p* > 0.05 each, two-way ANOVA). (C) No significant differences in wildtype and GluN2D KO mice in thermal nociception assessed by the hot plate test (p > 0.05, unpaired t-test). (D) GluN2C KO mice exhibited significantly higher paw withdrawal thresholds compared to wildtype at several time points post-CFA injection (post 6 h ***p = 0.0009, 24 h ***p = 0.0008, 48 h ****p < 0.0001, 72 h ***p = 0.0006, 96 h **p = 0.0014, 120 h *p = 0.0107, 169 h *p = 0.0176). (D′) No change in mechanical threshold in von Frey analysis in left (non-CFA injected paw) paw. (E, E’) GluN2C KO mice showed significantly lower licking behavior compared to wildtype in delayed phase of formalin-induced acute pain condition (*p < 0.05, two-way ANOVA). (F) A significant reduction in paw withdrawal latency was observed in GluN2C KO mice compared to wildtype (***p = 0.0002, unpaired *t*-test). Data were analyzed by one-way or two-way ANOVA or unpaired t-test wherever appropriate. Each data point represents mean ± S.E.M.

We next tested whether GluN2D KO are also resistant to mechanical allodynia in inflammatory pain using complete Freund’s adjuvant (CFA) and formalin model. In both inflammatory pain models, no significant differences in the von Frey responses to CFA (Fig. 2A (right paw), 2A’ (uninjected left paw)) and formalin (Fig. 2B, B’) were observed between wildtype and GluN2D KO group (*P* > 0.05, two-way ANOVA each). In the hot plate test, there was no significant difference in response to noxious heat stimuli between wildtype and GluN2D KO mice (Fig. 2C, *P* > 0.05, unpaired *t*-test). We next evaluated GluN2C KO mice in inflammatory pain models. In CFA model GluN2C KO showed higher paw withdrawal threshold compared to wildtype (post 6 h *p* = 0.0009, 24 h *p* = 0.0008, 48 h *p* < 0.0001, 72 h *p* = 0.0006, 96 h *p* = 0.0014, 120 h *p* = 0.0107, 169 h *p* = 0.0176, Fig. 2D). Two-way ANOVA showed a significant effect of genotype (F (1, 8) = 26.94, p = 0.0008), interaction (F (7, 55) = 3.785, *p* = 0.0020), and time (F (7, 55) = 99.47, p < 0.0001).

In addition, GluN2C KO mice had lower paw licking response in delayed phase of formalin test (at 45 mins *p* = 0.0344, Fig. 2E, at delay phase *p* < 0.05 Fig. 2E’). Two-way ANOVA showed a significant effect of genotype (F (1, 38) = 4.337, *p* = 0.0441), and time (F (1, 38) = 66.85, p < 0.0001). These results suggest that GluN2C subunit partially contribute to mechanical allodynia in inflammatory pain. Interestingly, GluN2C KO mice had lower paw withdrawal latency in the hot plate test (*p* = 0.0002, unpaired *t*-test, Fig. 2F). Overall, these results demonstrate that GluN2D-containing NMDARs are critical in the regulation of CINP but not in inflammatory pain whereas GluN2C subunit contributes to inflammatory pain and basal thermal sensitivity but not CINP demonstrating subunit-specific roles.

### 2.2. Deletion of GluN2D subunit in PV interneurons blocks development of cisplatin-induced mechanical hypersensitivity

**Fig. 3.**
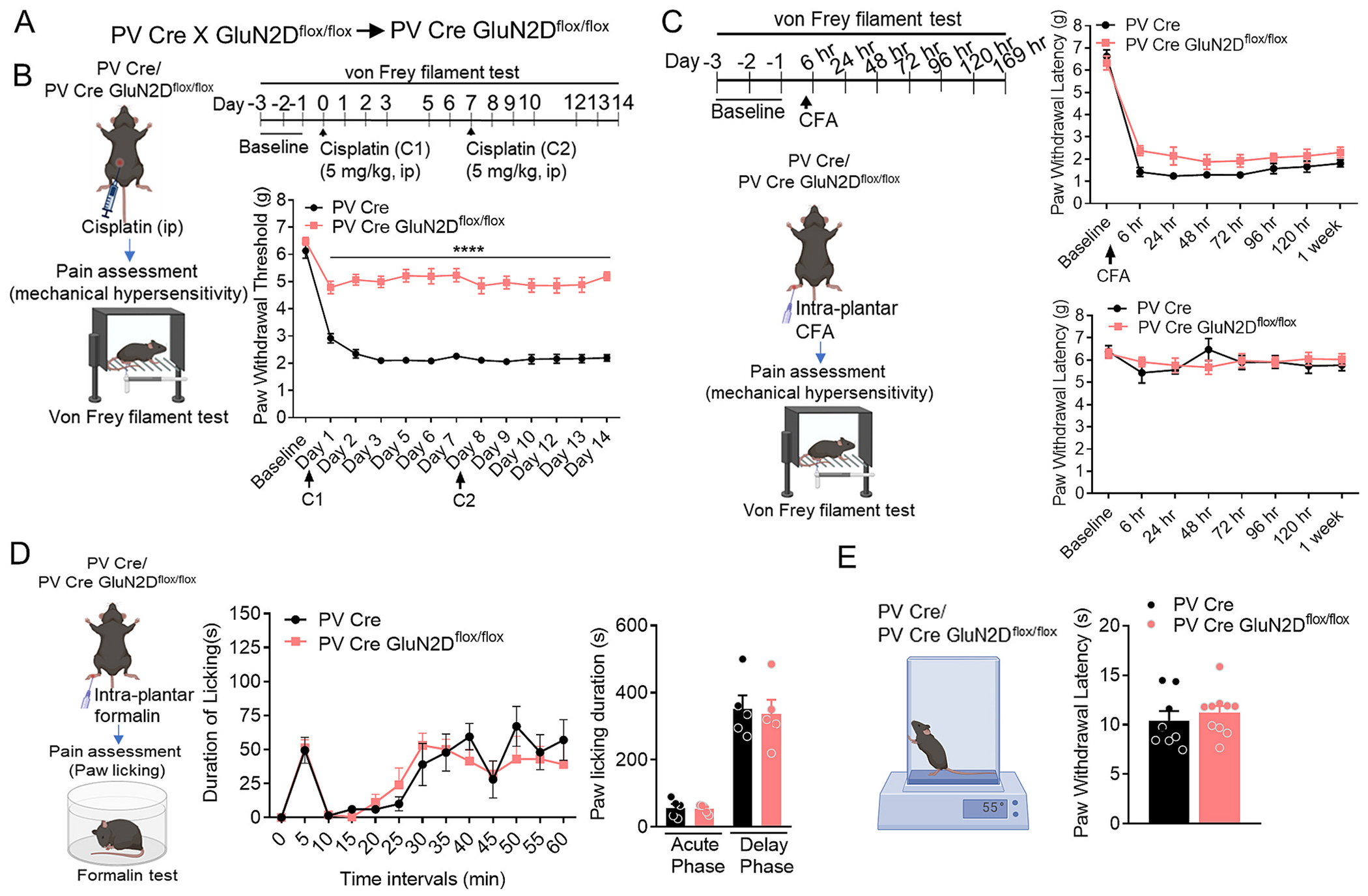
Role of GluN2D-containing NMDA receptors in parvalbumin interneurons (PVI) in the regulation of cisplatin-induced mechanical hypersensitivity. (A) Schematic illustration of the conditional deletion of the GluN2D subunit in PV interneurons. GluN2Dflox/flox mice were crossed with PV-Cre mice to generate PV-GluN2D KO mice. (B) Experimental design for testing PV-GluN2D KO mice in the cisplatin-induced neuropathic pain (CINP) model. Assessment of PV-GluN2D KO and PV-Cre mice in CINP. PV-GluN2D KO mice exhibited significantly higher paw withdrawal thresholds compared to PV-Cre mice throughout the entire testing period (****P < 0.0001 for each day; Two way ANOVA Bonferroni’s test). (C-E) Assessment of inflammatory pain and thermal nociception in PV-GluN2D KO and control PV-Cre mice. No significant differences were observed in CFA-induced mechanical hypersensitivity (*P* > 0.05, C), formalin-induced pain responses (P > 0.05, D), and hot plate test (P > 0.05, E). Data were analyzed by unpaired *t*-test or two-way ANOVA wherever appropriate. Each data point represents mean ± S.E.M.

Knowing that, the GluN2D-containing NMDARs are enriched in interneurons and particularly PVIs (Alsaad et al., 2019; Gawande et al., 2023a; Perszyk et al., 2016; von Engelhardt et al., 2015), we next tested whether GluN2D-containing NMDARs in PVIs regulate sensitivity to CINP. To achieve our goal, we conditionally deleted GluN2D subunit from PVIs by crossing GluN2D^flox/flox^ mice with PV-Cre mice (referred to hereafter as PV-GluN2D KO mice) as previously described (Liu et al., 2021; Gawande et al., 2023a) (Fig. 3A). We have previously characterized this model, demonstrating reduction in GluN2D expression in the cortex and other brain regions (Gawande et al., 2023a, 2023b). We tested PV-GluN2D KO mice in CINP. Similar to the observations in GluN2D KO mice, PV-GluN2D KO mice were found to have significantly higher paw withdrawal threshold compared to control PV-Cre mice (Fig. 3B). The effect was observed during the entire duration days 1–14 of von Frey analysis (*p* < 0.0001 each; Bonferroni’s test, Fig. 3B). Two-way ANOVA showed a significant effect of genotype (F (1, 181) = 1127, p < 0.0001), interaction (F (12, 181) = 7.168, p < 0.0001), and time (F (12, 181) = 29.50, p < 0.0001). No significant differences were observed in CFA-induced (*p* > 0.05) and formalin-induced inflammatory pain conditions (p > 0.05) and in the hot plate test (p > 0.05, Fig. 3C-E) thus suggesting that GluN2D-containing NMDAR in PVIs exclusively regulate CINP but not acute and inflammatory pain.

### 2.3. GluN2D-containing NMDAR in nucleus accumbens gates mechanical hypersensitivity in the cisplatin-induced neuropathic pain

**Fig. 4.**
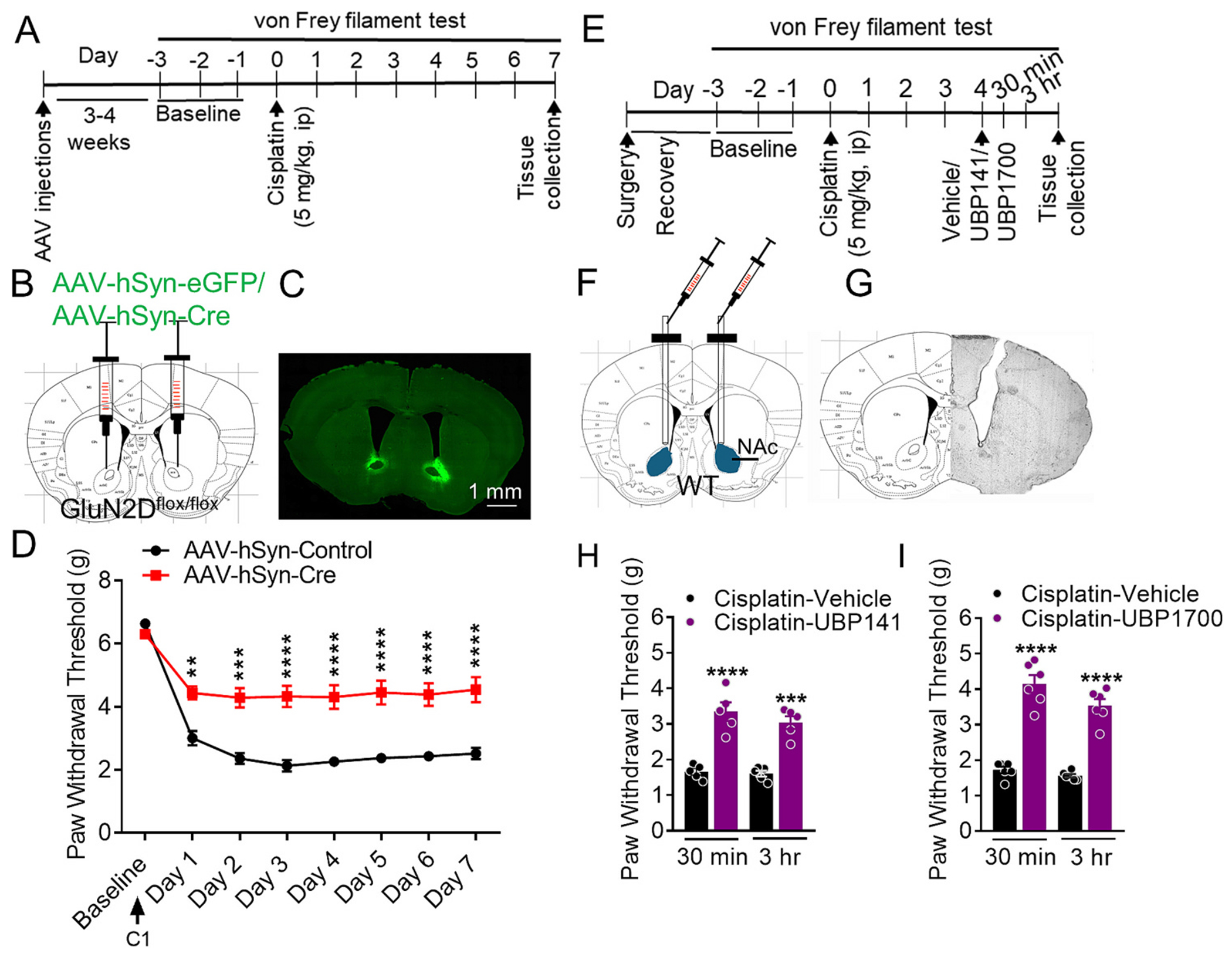
Involvement of GluN2D-containing NMDA receptors in the NAc in cisplatin-induced neuropathic pain. (A) Experimental design. (B) Local deletion of GluN2D in the nucleus accumbens (NAc) using AAV constructs. Schematic showing injection of either AAV-hSyn-eGFP or AAV-hSyn-Cre into the NAc of the GluN2Dflox/flox mice (C) Representative image for site verification of AAV injection. (D) Paw withdrawal thresholds in response to mechanical stimulation were recorded daily for 7 days post-CINP induction. Mice with GluN2D deletion in the NAc (AAV-Cre) showed significantly higher paw withdrawal thresholds compared to control mice (AAV-eGFP) on day 1 (**p < 0.0064), day 2 (***p = 0.0001), and days 3–7 (****p < 0.0001 each; two-way ANOVA Bonferroni’s test, *n* = 9 each group). (E) Experimental timeline for assessment of effect of intra-NAc GluN2D antagonist on CINP. (F) Mice underwent cannulation surgery for the implantation of bilateral cannulas in the NAc. (G) Representative image of cannula verification showing cannula placement in the NAc. (H–I) Effect of selective GluN2D antagonist UBP141 and UBP 1700 on mechanical hypersentivity in CINP model. Intra-NAc injections of UBP141 significantly increased paw withdrawal thresholds (30 min: ****p < 0.0001, and 3 h: ***p = 0.0001, *n* = 5 each group) compared to vehicle treatment. Similarly, UBP1700 treatment led to a significant increase in paw withdrawal threshold at both 30 min and 3 h time points (****p < 0.0001, *n* = 6 each group). Data were analyzed by one-way or two-way ANOVA wherever appropriate. Each data point represents mean ± S.E.M.

We next explored the potential brain region where GluN2D-containing receptors may produce effect on neuropathic pain. We focused on supraspinal brain regions. Recent studies have established a role of nucleus accumbens (NAc) in pain-related behaviors (Benarroch and Case, 2016; Harris and Peng, 2020; Yang et al., 2020) with a potential role of GluN2D subunit (Jing et al., 2022). Thus we assessed the impact of selective ablation of GluN2D subunit from neurons in the NAc on CINP. GluN2D^flox/flox^ mice received stereotaxic injections of AAV-hSyn-Cre (AAV-Cre) or AAV-hSyn-eGFP (AAV-control) into the NAc (Fig. 4A–C). Following an interval of 3–4 week for AAV expression and GluN2D ablation period, these mice underwent testing for CINP. We found that mice with GluN2D deletion in the NAc exhibited significant resistance to the development of neuropathic pain compared to controls (injected with AAV-eGFP), as evidenced by significantly higher paw withdrawal thresholds on day 1 (*p* < 0.0064), day 2 (*p* = 0.0001), and days 3–7 (*p* < 0.0001 each; Bonferroni’s test, Fig. 4D). Two-way ANOVA demonstrated significant effects of treatment (F (1, 64) = 134.2, p < 0.0001), interaction (F (7, 64) = 4.284, *p* = 0.0006), and time (F (7, 64) = 45.32, p < 0.0001). These results demonstrate that GluN2D-containing NMDARs within the NAc play a regulatory role in CINP.

We then used a pharmacological approach to examine the role of GluN2D subunit in the NAc in neuropathic pain. We tested the effect of intra-NAc injection of UBP141 and UBP1700 on mechanical hypersensitivity in CINP model. Mice were stereotaxically implanted with bilateral cannulas (Fig. 4F). After recovery from surgery mice were tested in CINP model. The intracranial dosage of UBP141 (10 μg per side) was based on the comparison of IC_50_ values with DQP1105, which is also a GluN2C/GluN2D preferring drug (Acker et al., 2011), which we have previously used for intracranial NAc experiments (Shelkar et al., 2022). The highly potent UBP1700 with nanomolar affinity (Wang et al., 2020) in comparison to UBP141 was used at 70 ng/side. A robust increase in the paw withdrawal threshold was observed following the administration of UBP141 (10 μg/side, 30 min: p = 0.0001, 3 h: *p* = 0.0003, Bonferroni’s test, Fig. 4H) and UBP1700 (70 ng/side, p < 0.0001 at both 30 min and 3 h time points, Bonferroni’s test, Fig. 4I). One-way ANOVA showed significant effect of UBP 141 (F (3, 16) = 29.01, p < 0.0001) and UBP1700 (F (3, 20) = 62.39, p < 0.0001) treatments on mechanical hypersensitivity in CINP.

### 2.4. Accumbal GluN2D-containing NMDARs do not affect reward behavior

**Fig. 5.**
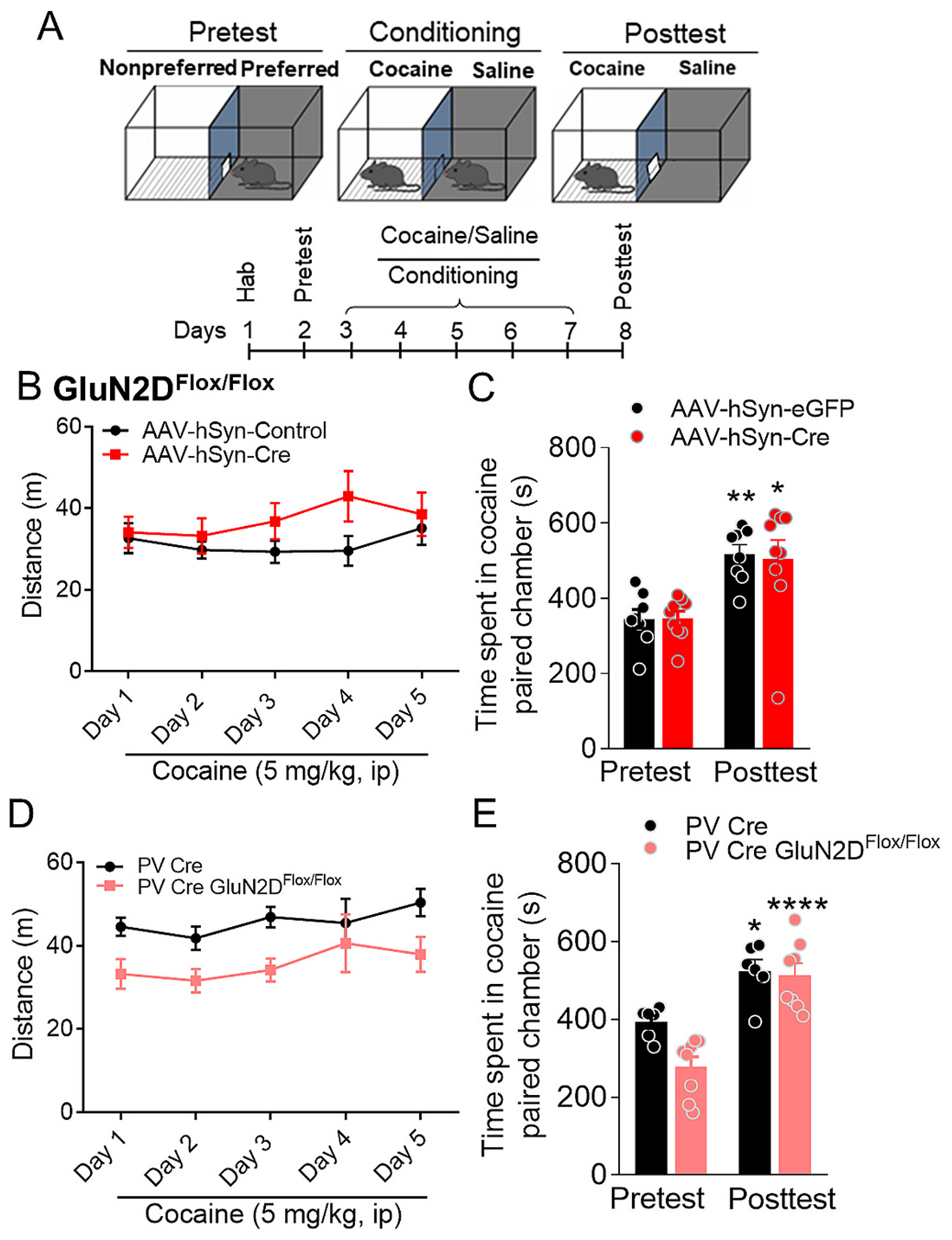
Effect of GluN2D deletion in NAc on cocaine-induced conditioned place preference. (A) Experimental timeline depicting the schedule of CPP test. (B, C) GluN2D^flox/flox^ mice injected with AAV-hSyn-eGFP (AAV-control, *n* = 8) or AAV-hSyn-cre (AAV-Cre, *n* = 10) in NAc were tested in cocaine CPP. No significant differences in cocaine-induced sensitization (change in locomotor activity upon repeated injection) (*p* > 0.05, two-way ANOVA, Bonferroni’s test). Following cocaine conditioning both AAV-control and AAV-Cre injected mice showed significant increase in time spent during posttest in cocaine-paired chamber (**p = 0.008, *p = 0.0112 vs respective pretest), however, no significant differences were observed between the groups (P > 0.05, one-way ANOVA, Bonferroni’s test). (D, E) PV-Cre (n = 6) and PV-GluN2D KO (n = 8) mice were tested in cocaine CPP. No significant differences in cocaine-induced sensitization (p > 0.05, two-way ANOVA). The total distance traveled after cocaine injection was significantly lower in PV-GluN2D KO mice (two-way repeated measures ANOVA, F (1, 12) = 5.552, genotype factor, *p* = 0.036). Following cocaine conditioning both PV-Cre and PV-GluN2D KO mice showed significantly increased time spent in cocaine-paired chamber during posttest (*p = 0.0275, ****p < 0.0001 vs respective pretest), however, no significant differences were observed in between the groups (P > 0.05, one-way ANOVA, Bonferroni’s test). Data were analyzed by one-way or two-way ANOVA wherever appropriate. Each data point represents mean ± S.E.M.

Given the pivotal role of NAc in reward processing, we next examined whether deletion of GluN2D subunit from the NAc on reward behavior. We used the cocaine-induced conditioned place preference (CPP) paradigm for these studies. GluN2D^flox/flox^ mice were injected with either AAV-Cre or AAV-control and after sufficient period for expression were tested for cocaine-induced CPP as previously described (Shelkar et al., 2022). No significant change in cocaine conditioning were observed upon ablation of GluN2D from NAc (*p* > 0.05, one-way ANOVA, Bonferroni’s test, Fig. 5B–C). We also tested PV-GluN2D KO mice in cocaine CPP paradigm. PV-GluN2D KO mice did not have altered cocaine-induced CPP (p > 0.05, one-way ANOVA, Bonferroni’s test, Fig. 5D–E). Interestingly, analysis of locomotor activity during cocaine injection session showed lower total distance traveled by PV-GluN2D KO (two-way repeated measures ANOVA, F (1,12) = 5.552, genotype factor, *p* = 0.036), suggesting lesser cocaine-induced psychotomimetic effect in this model. Because GluN2D KO do not show change in cocaine-induced hyperactivity (Shelkar et al., 2022), this result suggests PVI-specific role of GluN2D subunit. Also relevant to this observation is the role of GluN2D in NMDA channel blocker-induced psychotomimetic activity (Sapkota et al., 2016). Overall, these results suggest a differential involvement of GluN2D receptors in the NAc in modulating reward-related behaviors compared to nociceptive processing.

### 2.5. Cisplatin treatment increased excitatory neurotransmission in wildtype mice but not in PV-GluN2D KO mice

**Fig. 6.**
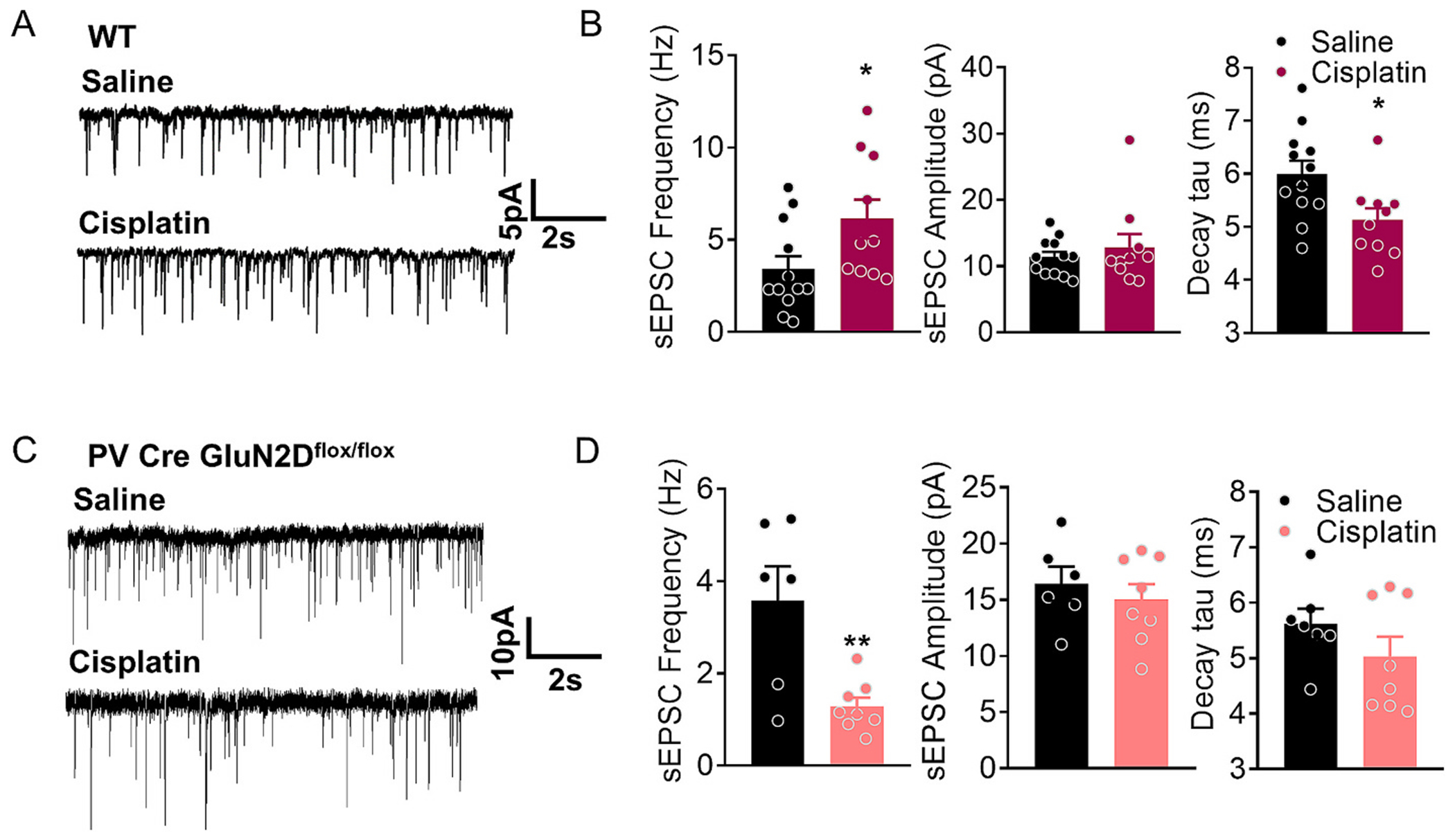
Effects of cisplatin treatment on excitatory neurotransmission in nucleus accumbens. (A) Whole-cell voltage-clamp recordings from NAc in saline and cisplatin treated wildtype mice was conducted. Spontaneous excitatory postsynaptic currents (sEPSCs) were obtained at a holding potential of −70 mV in the presence of picrotoxin. Downward deflections represent sEPSC events. (B) In wildtype mice cisplatin treatment significantly increased sEPSC frequency (*p = 0.0387, unpaired *t*-test) and decreased decay time compared to saline-treated mice (*p = 0.0182, unpaired t-test). No significant change in sEPSC amplitude was noted (*n* = 12 saline, 10 cisplatin neurons from *n* = 3 mice each group). (C) Whole-cell voltage-clamp recordings from NAc in PV-GluN2D KO mice was conducted and sEPSC events were recorded. (D) Cisplatin treatment significantly reduced sEPSC frequency compared to saline-treated mice (**p = 0.005, unpaired t-test). No significant change in sEPSC amplitude and decay time was noted (*n* = 7 (saline), 8 (cisplatin) neurons from n = 3 mice each group). Data were analyzed by unpaired t-test. Each data point represents mean ± S.E.M.

**Fig. 7.**
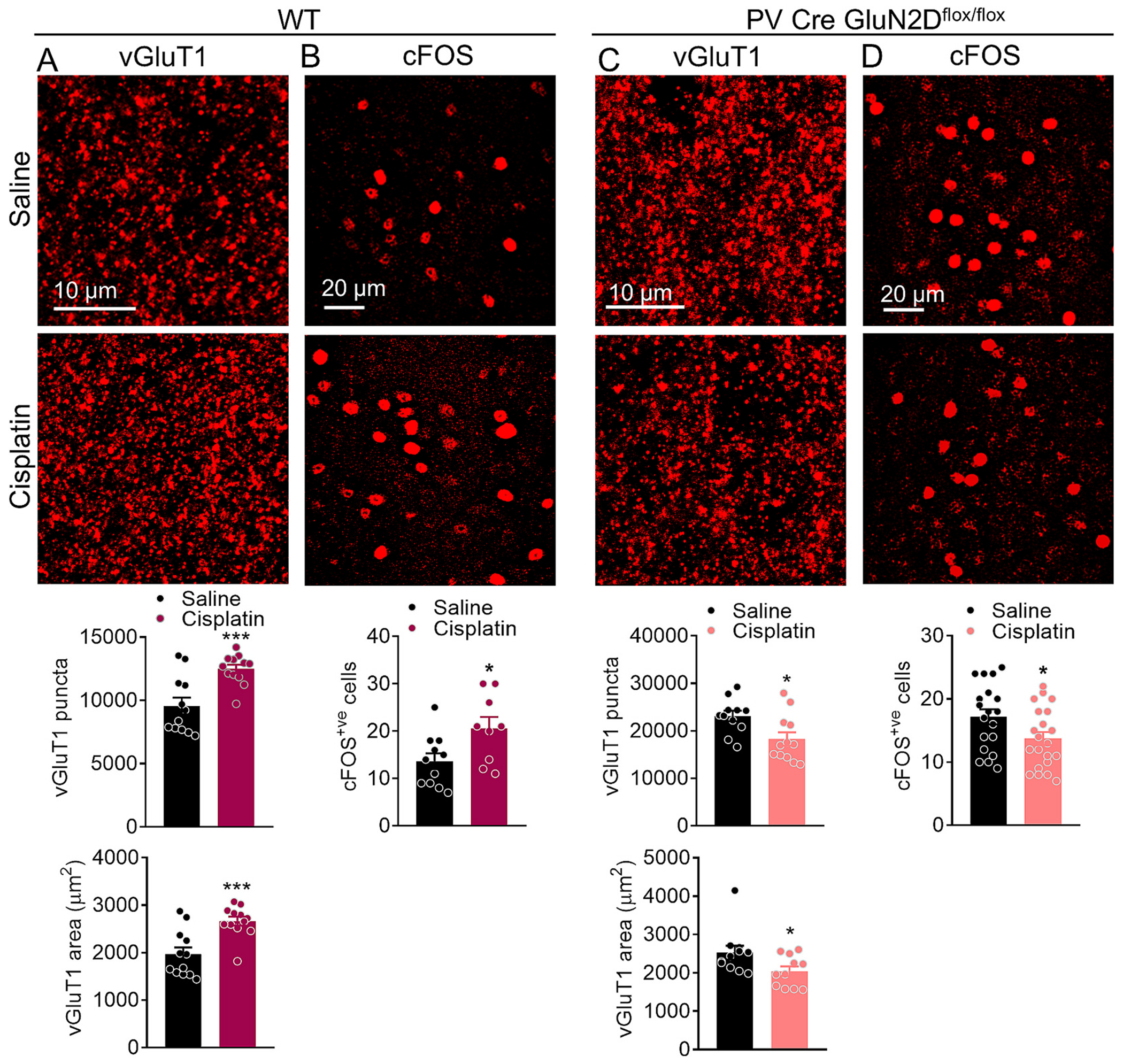
Cisplatin treatment increased NAc neuronal activation (c-Fos) and cortical (vGluT1) projections to NAc in wildtype mice but not in PV-GluN2D KO mice. Representative images from NAc of saline and cisplatin treated wildtype mice immunostained for vGluT1 (A), and c-Fos (B) and PV-GluN2D KO mice immunostained for vGluT1 (C), and c-Fos (D). (A) A significant increase in vGluT1 puncta (***p = 0.0007, unpaired t-test) and area (***p = 0.0005, unpaired t-test) was observed in cisplatin-treated mice as compared to the saline-treated wildtype mice. (B) A significant increase in c-Fos + ve cell (*p = 0.0253) in cisplatin treated mice compared to saline treatment. (C) A significant reduction in vGluT1 puncta (*p = 0.0154, unpaired t-test) and area (*p = 0.0370, unpaired t-test) was observed in cisplatin treated mice as compared to the saline treated PV-GluN2D KO mice. (D) A significant reduction in c-Fos + ve cell (*p = 0.0349) in cisplatin treated mice compared to saline treatment. Data were analyzed by unpaired t-test. Data from n = 5 mice each group were collated and analyzed. Each data point represents mean ± S.E.M.

We next investigated potential changes in excitatory neurotransmission in NAc neurons that may account for CINP. Whole-cell voltage-clamp recordings at a holding potential of −70 mV in the presence of picrotoxin were obtained in a random manner from MSNs identified based on morphology and abundance as we have previously described (Liu et al., 2020; Gawande et al., 2021). Downward deflections representing sEPSC events were recorded and analyzed. Wildtype mice treated with cisplatin, showed a significant increase in excitatory neurotransmission within NAc neurons, as evidenced by a significant increase in sEPSC frequency (*p* = 0.0387, unpaired *t*-test, Fig. 6B). Notably, in the PV-GluN2D KO mice, we found a significant reduction in the sEPSC frequency (*p* = 0.005, unpaired t-test, Fig. 6D) following cisplatin treatment as compared to the saline-treated mice. No change in the sEPSC amplitude was observed following saline or cisplatin treatments in both genotypes. Decay time was significantly decreased (*p* = 0.0182, unpaired *t*-test) in wildtype mice treated with cisplatin as compared to saline, whereas there was no change in PV-GluN2D KO mice. These results suggest a crucial role for GluN2D-containing NMDAR in PVIs in regulating neuroplastic changes in the context of CINP in the NAc. We also compared basal effect of GluN2D ablation from PVIs. No change in the sEPSC frequency between wildtype and PV-GluN2D KO mice was observed. However, the amplitude of sEPSCs was significantly higher in PV-GluN2D KO (p = 0.005, unpaired t-test). We next evaluated whether there are changes in excitatory projections and neuronal activity in the NAc in CINP. Immunohistochemistry was conducted for markers for excitatory cortical and thalamic projections i.e. vGluT1 and vGluT2, respectively (Fremeau Jr et al., 2001). A significant increase in vGluT1 puncta (puncta: *p* = 0.0007, area: *p* = 0.0005, unpaired t-test, Fig. 7A) in the NAc was observed in CINP. Furthermore, an increase in the number of c-Fos positive neurons was observed in the NAc of CINP model (*p* = 0.0253, unpaired t-test, Fig. 7B). On the contrary, a significant reduction in vGluT1 puncta (puncta: *p = 0.0154, area *p = 0.0370, unpaired t-test, Fig. 7C) and c-Fos positive cells (*p = 0.0349, Fig. 7D) was observed in cisplatin treated mice as compared to the saline treated PV-GluN2D KO mice. Interestingly, higher basal number of vGluT1 puncta was observed in PV-GluN2D KO mice. Although we did not find a change in frequency, the amplitude of sEPSCs in PV-GluN2D KO was significantly higher. We do not fully understand the reasons for these changes, however based on our previous analysis of PV-GluN2D KO in the medial prefrontal cortex (Gawande et al., 2023a, 2023b), it is possible that there is higher baseline excitatory tone in the PV-GluN2D KO. No change in the vGluT2 puncta or area were observed (*p* > 0.05, unpaired *t*-test, Supplementary Fig. 1) Together these results demonstrate an increase in excitatory neurotransmission mediated by cortical inputs in the NAc in the CINP model, whereas a reduction in PV-GluN2D KO mice.

### 2.6. Chemogenetic inhibition of PVIs in the NAc reduces mechanical hypersensitivity in CINP model

**Fig. 8.**
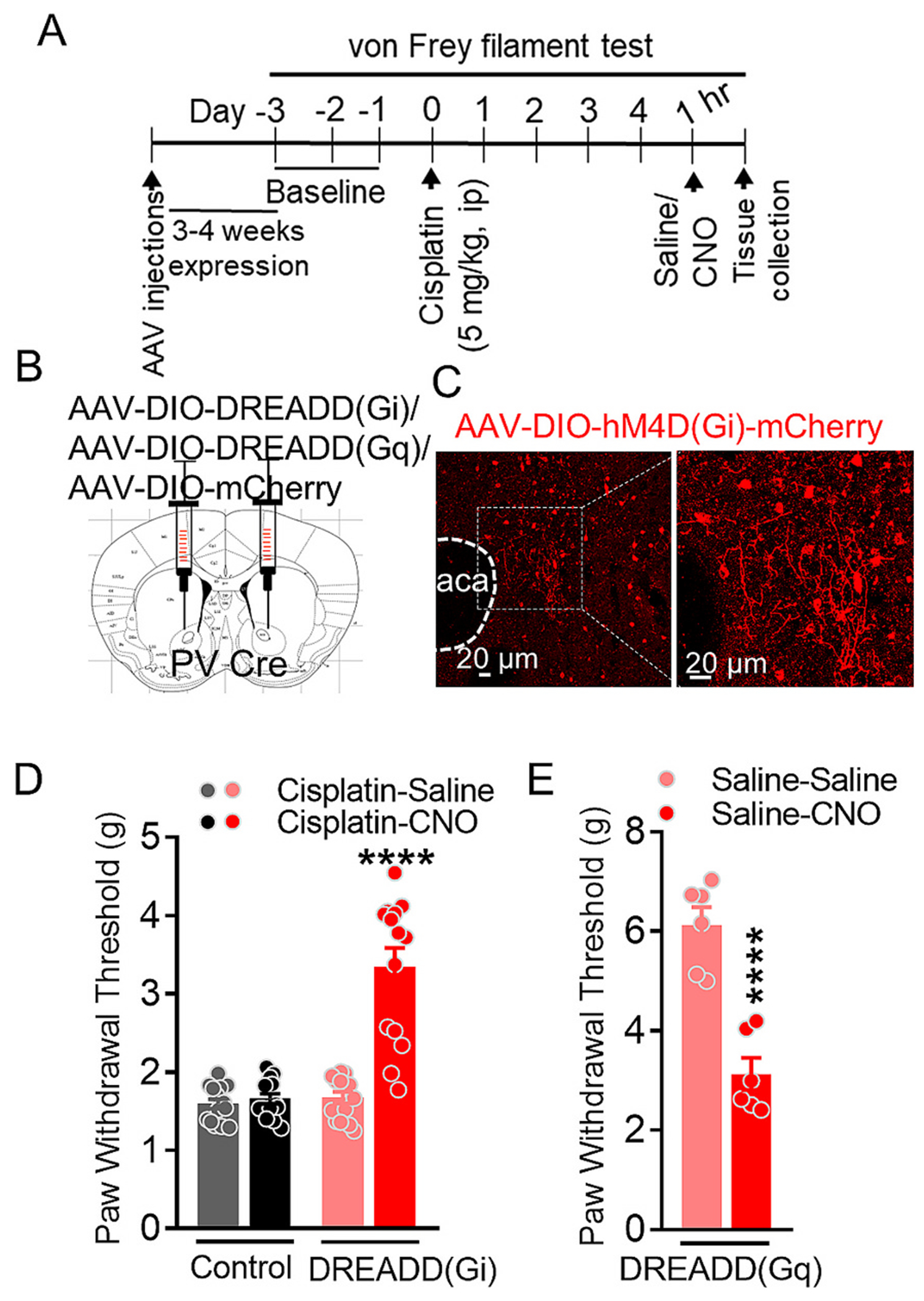
Chemogenetic inhibition of PVIs in NAc alleviated CINP. (A) Experimental design. (B) Schematic showing site of injection of AAV-Syn-DIO-hM4D (Gi)-mCherry (DREADD(Gi)) or AAV-Syn-DIO-hM3D(Gq)-mChreyy (DREADD (Gq)) or AAV-DIO-mCherry in the nucleus accumbens (NAc) of the PV-Cre mice. (C) Representative image for site verification of AAV injection. (D) Paw withdrawal thresholds in response to mechanical stimulation were recorded daily during baseline and post-CINP induction. Following cisplatin treatment DREADD (Gi) were treated with saline or CNO. CNO treatment in these mice showed significant increase in paw withdrawal threshold compared to the saline treatment (****p < 0.0001; one-way ANOVA, Bonferroni’s test, *n* = 14 each group). No significant effect of saline vs CNO treatment on paw withdrawal threshold in AAV-DIO-mCherry injected mice (p > 0.05). (E) A significant reduction in paw withdrawal threshold following CNO treatment in saline treated DREADD(Gq) mice (p < 0.0001, unpaired t-test, n = 6 each group). Data were analyzed by one-way ANOVA or unpaired t-test wherever appropriate. Each data point represents mean ± S.E.M.

We next tested whether chemogenetic inhibition of PVIs in the NAc could rescue mechanical allodynia in CINP. PV-Cre mice were injected with AAV-Syn-DIO-hM4D(Gi)-mCherry into the NAc to specifically express inhibitory DREADD receptors in PVIs. Following recovery, the mice were treated with cisplatin. After the development of mechanical hypersensitivity, as indicated by a significant reduction in paw withdrawal threshold, the mice received either saline or CNO (1 mg/kg, i.p.). One hour post-injection, the mice were subjected to the von Frey filament test. CNO treatment significantly increased the paw withdrawal threshold (*p* < 0.0001, one-way ANOVA, Bonferroni’s test, Fig. 8D), indicating an alleviation of mechanical hypersensitivity. One-way ANOVA revealed a significant effect of CNO on paw withdrawal latency (F (3, 52) = 39.81, p < 0.0001). In contrast, no significant differences were observed between saline and CNO treatment in mice injected with AAV-Syn-DIO-mCherry (p > 0.05, one-way ANOVA, Bonferroni’s test). These findings suggest that inhibition of PVIs in the NAc alleviates CINP-like mechanical hypersensitivity.

To explore whether activation of PVIs in the NAc could exacerbate pain responses, we injected PV-Cre mice with AAV-DIO-hM3D(Gq)-mCherry into the NAc. After recovery, these mice received saline instead of cisplatin and, four days later, were treated with either saline or CNO. Notably, CNO treatment led to a significant reduction (p < 0.0001, unpaired t-test, Fig. 8E) in the paw withdrawal threshold, indicating the induction of a pain-like state. In conclusion, these results demonstrate that chemogenetic inhibition of PVIs in the NAc alleviates neuropathic pain-like conditions, while activation of PVIs promotes pain-like behaviors, underscoring the role of NAc PVIs in modulating pain sensitivity.

## 3. Discussion

We have demonstrated the subunit-specific roles of GluN2D- and GluN2C-containing NMDARs in the selective regulation of pain transmission across different pain conditions. These include CINP, CFA- or formalin-induced inflammatory pain, and thermal nociception. Our study provides convincing evidence that GluN2D-containing NMDARs play a critical role in the development and regulation of CINP, but not in inflammatory or thermal pain, highlighting subunit-specific functions in different pain modalities.

Using genetic knockout models, we showed that GluN2D knockout mice were resistant to the development of CINP but showed no differences in sensitivity to CFA- or formalin-induced inflammatory pain. This aligns with previous findings reporting GluN2D subunit deletion prevented the development of tactile allodynia after partial sciatic nerve ligation but does not contribute to formalin-induced inflammatory pain (Hizue et al., 2005). Furthermore, another study supports these observations, reporting that the local administration of PPDA, a selective GluN2C/2D subunits antagonist, produces antinociceptive effects in a spinal nerve ligation (SNL) model (Jing et al., 2022). Additionally, the therapeutic potential of modulating the GluN2D subtype for neuropathic pain is supported by other studies (Hildebrand et al., 2014; Dedek and Hildebrand, 2022). Along similar lines, we observed that systemic intraperitoneal administration of selective GluN2D antagonists UBP141 and UBP1700 significantly alleviated pain in the CINP model, reinforcing the importance of GluN2D in neuropathic pain regulation.

Interestingly, GluN2C knockout mice exhibited no change in sensitivity to CINP but displayed reduced sensitivity to CFA and increased sensitivity to formalin-induced pain and thermal nociception in hot plate test. While the precise mechanisms underlying these behavioral differences are not fully understood, it is likely that the cell type-specific expression patterns of GluN2D and GluN2C subunits play a significant role. Specifically, GluN2D is predominantly expressed in interneurons, while GluN2C is expressed in astrocytes in majority of corticolimbic regions including cortex and nucleus accumbens (Ravikrishnan et al., 2018; Shelkar et al., 2022; Gawande et al., 2023a). These differences in cellular localization could explain the distinct effects of GluN2C and GluN2D on different pain modalities. Indeed, astrocytes and their secreted factors such as thrombospondin (TSP) 1, TSP 4 and hevin have been shown to play a crucial role in regulating neuropathic and inflammatory pain conditions (Kim et al., 2012, 2016; Chen et al., 2022). Specifically, hevin KO mice showed faster recovery than wildtype mice in phase 2 but not phase 1 of formalin-induced spontaneous pain and carrageenan-induced inflammatory pain models (Chen et al., 2022). Sciatic nerve injury activates mGluR5 signaling in astrocytes, leading to increased Ca^2+^ transients and the release of thrombospondin 1 (TSP1), which promotes synapse formation and induces mechanical hyperalgesia. Blocking this pathway alleviates pain, underscoring the involvement of astrocytes in chronic pain maintenance (Kim et al., 2016). Nerve injury also upregulates TSP4 in spinal astrocytes, facilitating neuropathic pain by modulating excitatory synaptic transmission (Kim et al., 2012). Given that GluN2C regulates secretion of astrocytic synaptogenic factors (Shelkar et al., 2022), our findings suggest that the differential expression of GluN2C and GluN2D subunits contributes to distinct pain pathways, highlighting the key role of astrocytes in the modulation of pain. Overall, our results highlight the distinct yet complementary roles of GluN2D- and GluN2C-containing NMDARs in the regulation of various pain conditions.

Knowing that GluN2D-containing NMDARs are highly enriched in interneurons, particularly in PVIs (Alsaad et al., 2019; Gawande et al., 2023a; Perszyk et al., 2016; von Engelhardt et al., 2015), we aimed to investigate whether these receptors play a role in regulating sensitivity to CINP. These neurons play a critical role in maintaining the balance between excitatory and inhibitory signals in the brain through feed-forward inhibition, thus orchestrating the coordinated rhythmic activity of primary neurons (Hu et al., 2014; Leitch, 2024). PV-positive neurons have been implicated in the development of hyperalgesia in mice experiencing fibromyalgia pain (Miyahara et al., 2021). However, the role of PV-positive neurons in the NAc in neuropathic pain remains unclear. To this end, we conditionally deleted the GluN2D subunit specifically from PVIs by crossing GluN2D^flox/flox^ mice with PV-Cre mice, generating the PV-GluN2D KO mouse line (Liu et al., 2021). This genetic approach allowed us to selectively target GluN2D in PVIs without affecting other neuronal populations, as confirmed by reduced GluN2D expression in the cortex and other relevant brain regions in this model (Gawande et al., 2023a, 2023b). Importantly, PV-GluN2D KO mice showed a significantly higher paw withdrawal threshold compared to control PV-Cre mice in the CINP model, indicating reduced sensitivity to CINP. Interestingly, PV-GluN2D KO mice did not exhibit any significant differences in pain sensitivity under CFA-induced inflammatory pain, formalin-induced pain, or acute thermal nociception indicating that GluN2D-containing NMDARs in PVIs are selectively involved in the regulation of neuropathic pain but do not influence responses to acute or inflammatory pain stimuli. Our findings suggest that GluN2D-containing NMDARs in PVIs are exclusively responsible for modulating CINP, a conclusion supported by the specificity of GluN2D deletion to this pain modality.

The NAc, a key component of the mesolimbic reward system, has extensive cortical connections, implicating its role in various functions, including pain processing (Benarroch and Case, 2016; Harris and Peng, 2020; Yang et al., 2020). Dysfunction of NMDARs in the NAc has been linked to several neurological and psychiatric disorders, including neuropathic pain. Notably, an increase in currents sensitive to GluN2C/2D-containing NMDARs modulators is observed after SNL, and pharmacological inhibition of these NMDAR subunits in the NAc effectively alleviated SNL-induced mechanical allodynia and depressive-like behaviors (Jing et al., 2022). In line with this, we found that conditional ablation of GluN2D-containing NMDARs in the NAc or selective pharmacological inhibition of these receptors using a novel, potent, and highly selective antagonist, significantly prevented the development of neuropathic pain-like condition. Notably, conditional deletion of GluN2D from NAc, did not produce any changes in the reward-related behaviors. These findings underscore the critical role of GluN2D-containing NMDARs in the NAc in regulating neuropathic pain, while sparing the reward circuitry, making them a promising target for pain therapy without affecting reward mechanisms.

Studies have also shown that the NAc becomes activated in response to pain-predictive cues (Baliki et al., 2010) and that chronic pain leads to an increase in cFos activation within the NAc. Additionally, specific populations of NAc neurons exhibit heightened activity during pain, with the selective activation of these neurons intensifying pain-like conditions (Sun et al., 2023). In line with these findings, our study revealed that cisplatin administration significantly increased cFos-positive cells in the NAc of wildtype mice, while PV-GluN2D KO mice showed a marked reduction in cFos-positive cells. Additionally, in concurrence with the cFos results, we observed a significant increase in the frequency of sEPSC in wildtype MSNs, contrasting with a decrease in sEPSC in PV-GluN2D KO mice. These results demonstrates a role of GluN2D in modulating pain-related neural activity in the NAc. Further supporting these observations, previous studies have highlighted that functional connectivity between the medial prefrontal cortex (mPFC) and the NAc is a strong predictor of pain persistence (Baliki et al., 2006, 2012). To examine potential changes in cortical input to the NAc following cisplatin treatment, we used vGluT1 labeling to assess alterations in excitatory neurotransmission. Our findings revealed a significant increase in vGluT1 immunoreactivity in the NAc of cisplatin-treated wildtype mice, while PV-GluN2D KO mice exhibited a marked decrease in vGluT1 labeling. These results further implicate GluN2D in mediating changes in cortical excitatory input to the NAc, underscoring its role in pain-related neuroplasticity. Interestingly, wildtype and PV-GluN2D KO showed opposite effects in excitatory neurotransmission in response to CINP. Similar opposite effect of CINP in wildtype and PV-GluN2D KO was also observed in the vGluT1 puncta count and c-fos labeling. Some conclusions can be drawn from this data. First, these findings suggest that cisplatin is still inducing neuroplastic changes, but these are not pain-driving changes in PV-GluN2D KO. In other words, cisplatin-induced neuroplasticity is not prevented by GluN2D loss but is reorganized. Secondly, since loss of GluN2D from PVIs results in a shift in excitatory-inhibitory balance (Gawande et al., 2023a, 2023b), it may be responsible for cisplatin producing opposite neuroplastic changes in PV-GluN2D KO. Indeed, the circuit changes produced by genotype X drug interaction may be complex and future studies are needed to address these issues.

Previous studies have demonstrated that PVIs in the anterior cingulate cortex (ACC) modulate neuropathic pain, such that optogenetic activation of PVIs increased, and their inhibition decreased, mechanical hypersensitivity in spared nerve injury mice (Zhang et al., 2015). In this study, we investigated the role of PVIs in the NAc in CINP, particularly focusing on GluN2D-containing NMDARs, which are predominantly expressed in PVIs. Using a chemogenetic approach, we employed Cre-dependent inhibitory DREADD to selectively inhibit PVIs in the NAc. Our findings revealed that chemogenetic inhibition of NAc PVIs significantly attenuated CINP, whereas animals expressing a control AAV in NAc PVIs exhibited no change in pain behavior following CNO administration. This confirmed the specificity of PVI inhibition in modulating pain responses. Additionally, chemogenetic activation of NAc PVIs in naïve mice led to a significant increase in mechanical hypersensitivity, further supporting the role of these interneurons in pain regulation. Given that GluN2D-containing NMDARs are predominantly expressed on PVIs, the data suggest that these receptors play a key role in modulating the activity of PVIs during pain states. Notably, selective inhibition or genetic ablation of GluN2D-containing NMDARs in NAc also prevented the development of neuropathic pain, without affecting reward-related behaviors. These results underscore the pivotal role of GluN2D-containing NMDARs on PVIs in the NAc in regulating neuropathic pain. While GluN2D receptors modulate pain processing without interfering with reward circuitry, PVIs themselves are critical for both the development and expression of neuropathic pain. We did not fully explore the circuitry from glutamatergic inputs to PVIs and their feedforward effect on MSNs that may contribute to CINP. However, some inferences can be drawn based on previous findings. Zhang et al., reported reduced excitability of pyramidal neurons because of an increase in feed-forward inhibition from PVIs in the cortex in neuropathic pain (Zhang et al., 2015). Accordingly, optogenetic inhibition of PVI reduced neuropathic pain. Conversely in normal animals, PVI excitation increased pain behaviors. EPSC or IPSC recordings were not obtained in this study. These observations are consistent with our findings that chemogenetic inhibition of PVIs in NAc or GluN2D deletion from PVIs reduced pain behaviors. In our studies we did not measure MSN excitability, but if a similar reduction in excitability of MSNs occurs, a homeostatic increase in excitatory inputs could be expected. This hypothesis will be consistent with our data showing an increase in sEPSC frequency in CINP. Thus, a likely scenario is opposite changes in excitability and excitatory neurotransmission in the MSNs in CINP which may be promoting pain. These questions need further assessment in future studies.

Although NMDARs have been implicated in several neurological and neuropsychiatric disorders, their therapeutic targeting has been challenging. Ketamine, a well-known NMDA channel blockers has been found to be effective in acute and chronic pain but whether this efficacy is mediated by subunit-specific effects remains to be determined. Nonetheless, several of ketamine’s behavioral effects have been attributed to GluN2D subunit (Sapkota et al., 2016). Our study uncovered a novel mechanism by which GluN2D subunit may provide analgesic effect and sets the stage for future studies to explore the role of GluN2D subunit as a therapeutic target for pain management.

## 4. Material and methods

### Animals:

We used wild-type, Grin2Dtm1a(EUCOMM)Wtsi (KO-first allele, GluN2D KO), Grin2Ctm1(EGFP/cre/ERT2)Wtsi (KO-first allele, GluN2C KO) mice (Wellcome Trust Sanger Institute). In addition, the GluN2D-flox/flox mice were generated by removing the reporter cassette by crossing Grin2Dtm1a(EUCOMM)Wtsi mice with B6-SJL-Tg(ACTFLPe) 9205Dym/J line. Conditional deletion of GluN2D from PVIs was achieved by crossing PV-Cre mouse line (Jax stock number 017320) with GluN2Dflox/flox mice as previously described (Gawande et al., 2023a). Mice at 8–10 weeks of age, weighing 20–30 g of both the sexes were used in the present study. Mice were housed at a constant temperature (22 ± 1 °C) and a 12-h-light/–dark cycle with ad libitum access to food and water. All animals were group housed except the mice implanted with guide cannula were housed singly to prevent fighting- or grooming-induced retraction of cannula. All procedures were approved by the Creighton University and Texas A&M Institutional Animal Care and Use Committee and conformed to the NIH Guide for the Care and Use of Laboratory Animals.

#### 4.1. Drugs

UBP 141 and UBP 1700 (Hello Bio Inc., Princeton, NJ, USA) were dissolved in water with 1 eq NaOH (50 mM). Cisplatin (cis-diammineplatinum(II) dichloride, Sigma-Aldrich, St. Louis, MO, USA), Cocaine HCl (Sigma, St. Louis, MO, USA), Clozapine-N-oxide (CNO; Hello Bio Inc., Princeton, NJ, USA) were dissolved in saline and injected intraperitoneally (ip). Sodium bicarbonate (4 %, Fischer chemical, Pittsburgh, PA) was injected subcutaneously (sc) before each cisplatin injection, Complete Freund’s adjuvant (CFA; 0.5 mg/mL heat-killed *M. tuberculosis*; Sigma, St. Louis, MO, USA) and formalin (5 % formalin) was injected into the hind paw.

#### 4.2. Pain induction models

##### 4.2.1. Cisplatin-induced neuropathic pain model

Mice were treated with saline or cisplatin and assessed for neuropathic pain (mechanical hypersensitivity) using the von Frey filament test. Cisplatin was injected (5 mg/kg, ip) once a week for two consecutive weeks to induce neuropathic pain-like condition. Cisplatin was dissolved in sterile 0.9 % saline and injected at a volume of 10 mL/kg of body weight. Prior to the cisplatin treatment, each mouse was treated subcutaneously with 1 mL of 4 % sodium bicarbonate to prevent nephrotoxicity-induced lethality (Guindon et al., 2014). The control group received an equivalent volume of saline (ip) in lieu of cisplatin. Pain induction and stability was monitored by testing behaviors each day using von Frey filament (IITC Life Sciences, Woodland Hills, CA).

##### 4.2.2. CFA inflammatory pain model

Inflammation was induced by intra-plantar injection of 10 μL of complete Freund’s adjuvant (0.5 mg/mL heat-killed *M. tuberculosis*) into the hind paw. Saline (pH 7.4) served as control. Mechanical sensitivity was measured using electronic von Frey filament at multiple time points (6 h to 1 week) after CFA injection.

##### 4.2.3. Formalin inflammatory pain model

The formalin (10 μL of 5 % formalin) was injected into the plantar surface of the right hind paw. The control mice were injected with 10 μL of saline. Each mouse was immediately placed in the observation chamber after injection, and the time licking the injected hind paw was recorded during the acute (0 to 15 min) and delay (15 to 45 min) phases.

#### 4.3. Behavioral assessment tests

##### 4.3.1. Von Frey filament test

Von Frey filament test in mice was performed using an electronic von Frey aesthesiometer (IITC systems) as described previously (Gandhi et al., 2021). Mice were habituated to custom testing chambers with a perforated bottom to provide access to their paws. Next, animals were habituated to the application of rigid filament (from IITC systems) applied perpendicularly to the plantar surface of the hind paw. Paw withdrawal threshold was measured as the force at which the animal showed nocifensive responses, such as brisk paw withdrawal, licking, or shaking of the paw. An average of 3 readings were taken for each time point.

##### 4.3.2. Hotplate test

The Hotplate test was performed using the IITC Hot plate analgesia meter as described previously (Gandhi et al., 2021). Briefly, mice were placed on the metal surface maintained at 55 °C enclosed by a plastic chamber and the response latency for paw licking or jumping behavior was recorded. The animal was removed from the hotplate after the nociceptive response or a cut-off latency of 20 s to prevent tissue damage.

##### 4.3.3. Cocaine-induced conditioned place preference (CPP) test

The CPP test was carried out as previously described (Shelkar et al., 2022) with some modification. The testing chambers consisting of two equal sized compartments (20 cm × 20 cm × 20 cm) with distinct contextual characteristics separated by a partition having a guillotine door was used for CPP study. On day 1, mice were habituated for 15 min with free access to both the chambers. On day 2, the baseline preference for each chamber was recorded for 15 min (pretest) to determine the baseline preference of each mouse to the one of the two chambers. After pretest readings were determined, cocaine was paired with the initial non-preferred chamber, and saline was paired with the preferred chamber. On the conditioning day, day 3, mice received an ip injection of cocaine (15 mg/kg, ip) and were restricted in non-preferred chamber for 30 min in morning. At least 5 h after the cocaine was injected, the same mice were injected with saline and placed in preferred chamber. The conditioning phase consisted of a total of 10 sessions held on five consecutive days, and each session was run for 30 min. On day 8, for post-test, mice were placed in the CPP box with the door open to have free access to both the chambers for 15 min in a drug free state and the time spent in each chamber was noted. Locomotor activity was recorded in the CPP box after cocaine injection during conditioning phase and analyzed using Anymaze and plotted.

#### 4.4. Cannulation and viral injection

Stereotaxic surgery was conducted as previously described (Shelkar et al., 2022) with minor modifications. For stereotaxic cannulation into the NAc, mice were anesthetized with isoflurane (NDC 66794–013-25, Piramal Critical Care, Bethlehem, PA, USA) and placed in a stereotaxic frame (51,733 U, Stoelting, Wood Dale, IL, USA). The skull was exposed, and a small hole was drilled through the skull and a 26-gauge stainless steel guide cannula was implanted bilaterally above the NAc at the stereotaxic coordinates (anteroposterior (AP): +0.9 mm, mediolateral (ML): ±1 mm, dorsoventral (DV): −4 mm; Paxinos and Franklin, 2001). The guide cannulae prepared in house were secured to the skull with stainless steel screws (PlasticOne, Roanoke, VA, USA) and dental acrylic cement (B1334, Ortho-Jet, Lang Dental, IL, USA). A stainless-steel dummy cannula was used to occlude the guide cannula when not in use. Experiments were conducted after 10 days of recovery from surgical procedure.

For virus injections, a small hole was drilled above the NAc (AP:+1.7 mm, ML: ±1 mm, DV: −4.2 mm). Virus particles AAV8.hSyn.eGFP and AAV8.hSyn.mCherry-Cre (University of Pennsylvania vector core) or the designer receptors exclusively activated by designer drug (DREADD) virus particles AAV-Syn-DIO-hM3D (Gq) or AAV-Syn-DIO-hM4D (Gi)-mCherry (Neurophotonics, Quebec, QC, Canada) were injected (100 nl) using a microliter syringe (NanoFil, World Precision Instruments, Sarasota, FL, USA) with a 33-gauge beveled needle (NF33BV-2, World Precision Instruments). The injection needle was lowered into the NAc core, and virus particles were delivered at a rate of 1 nl/s using a UMP3 microsyringe pump (World Precision Instruments). The needle was left in place at the injection site for an additional 10 min and thereafter slowly withdrawn over a period of 5 min. Incision was sealed with surgical tissue adhesive (3 M Vetbond Tissue Adhesive, MN, USA). Cannula and viral injection locations were verified after the end of behavioral experiments by examining the fixed brain tissue under a light or fluorescent microscope.

##### 4.4.1. Electrophysiology

Whole-cell electrophysiology was performed as previously described (Gawande et al., 2023a). After isoflurane anesthesia, mice were decapitated and brains were removed rapidly and placed in ice-cold artificial cerebrospinal fluid (aCSF) of the following composition (in mM): 130 NaCl, 24 NaHCO3, 3.5 KCl, 1.25 NaH2PO4, 2.4 CaCl2, 2.5 MgCl2 and 10 glucose saturated with 95 % O2/5 % CO2. 300 μm thick coronal sections were prepared using vibrating microtome (Leica VT1200, Buffalo Grove, IL, USA). Whole-cell patch recordings were obtained from neurons in NAc in voltage-clamp configurations with an Axopatch 200B (Molecular Devices, Sunnyvale, CA, USA). Glass pipettes with a resistance of 5–7 mOhm were used. Glass pipettes were filled with an internal solution consisting of 126 mM cesium methanesulfonate, 8 mM NaCl, 10 mM Hepes, 8 mM Na2-phosphocreatine, 0.3 mM Na2GTP, 4 mM MgATP, 0.1 mM CaCl2, and 1 mM EGTA (pH 7.3). QX-314 (2.9 mM) was added to block voltage-gated sodium channels in recorded cells. Picrotoxin was added to bath solution to isolate sEPSCs. Signal was filtered at 2 kHz and digitized at10 kHz using an Axon Digidata 1440 A analog-to-digital board (Molecular Devices, CA). Whole-cell recordings with a pipette access resistance less than 20 mOhm and that changed less than 20 % during the duration of recording were included.

##### 4.4.2. Immunohistochemistry

Immunohistochemistry was performed in control or PV-GluN2D KO mice treated with saline and cisplatin as previously described (Gandhi et al., 2021). Briefly, mice were transcardially perfused, and brains were removed and cryoprotected. The brains were cut in a coronal plane and sections passing through NAc were collected. Sections were washed with PBT (0.25 % Triton-X in 0.01 M PB) and incubated in appropriate blocking solution containing 10 % normal goat serum in PBT for 1 h at room temperature. Following blocking, sections were incubated in primary antibodies at appropriate concentrations (rabbit anti-vGluT1, 1:500, MABtechnology VGT1–3; rabbit anti-vGluT2, 1:500, MABtechnology VGT2–3; rabbit anti-cFOS, 1:500, Invitrogen PA%-143,600) in solution containing 5 % normal goat serum in PBT overnight at 4 °C. On the next day, sections were washed and thereafter incubated with the appropriate secondary antibodies conjugated to AlexaFluor 594 (goat anti-rabbit 1:500 in PBT) for 2 h at room temperature. Sections were then washed and mounted with Fluoromount-G (SouthernBiotech, Birmingham, AL, USA). Confocal images were acquired using a Nikon eclipse Ti2-E inverted confocal microscope with AX point scanner. Images of an equivalent region, 1024 pixels by 1024 pixels, were captured using a 60× oil immersion or 20× objectives at a 1× zoom. The NAc sections were scanned at 0.3 μm intervals along the z axis, and an optical section (7.2 μm thick) was taken from each tissue section. vGluT1 and vGluT2 puncta number and area were analyzed by NIS-Element image analysis software (Nikon, NY, USA). cFOS+ve cells were counted using ImageJ (NIH) software. Images from *n* = 5 mice per group were analyzed and plotted. Images were analyzed by a trained observer blind to genotypes.

#### 4.5. Statistical analyses

All data are presented as mean ± SEM. Data were analyzed using unpaired ***t***-test, or one-way or two-way ANOVA with post-hoc Bonferroni’s multiple comparisons test. Differences were considered significant if *p* < 0.05. Prism 8 (GraphPad Software Inc., San Diego, CA, USA) was used for analysis.

## Data Availability

The data supporting the findings of this study are available from the corresponding author upon reasonable request.
